# Supplementary figures and images for: Higher-level spatial prediction in natural vision across mouse visual cortex
Source: PLoS Comput Biol. 2026 Jan 20;22(1):e1013136. doi: 10.1371/journal.pcbi.1013136 (PMC12829946; doi:10.1371/journal.pcbi.1013136)

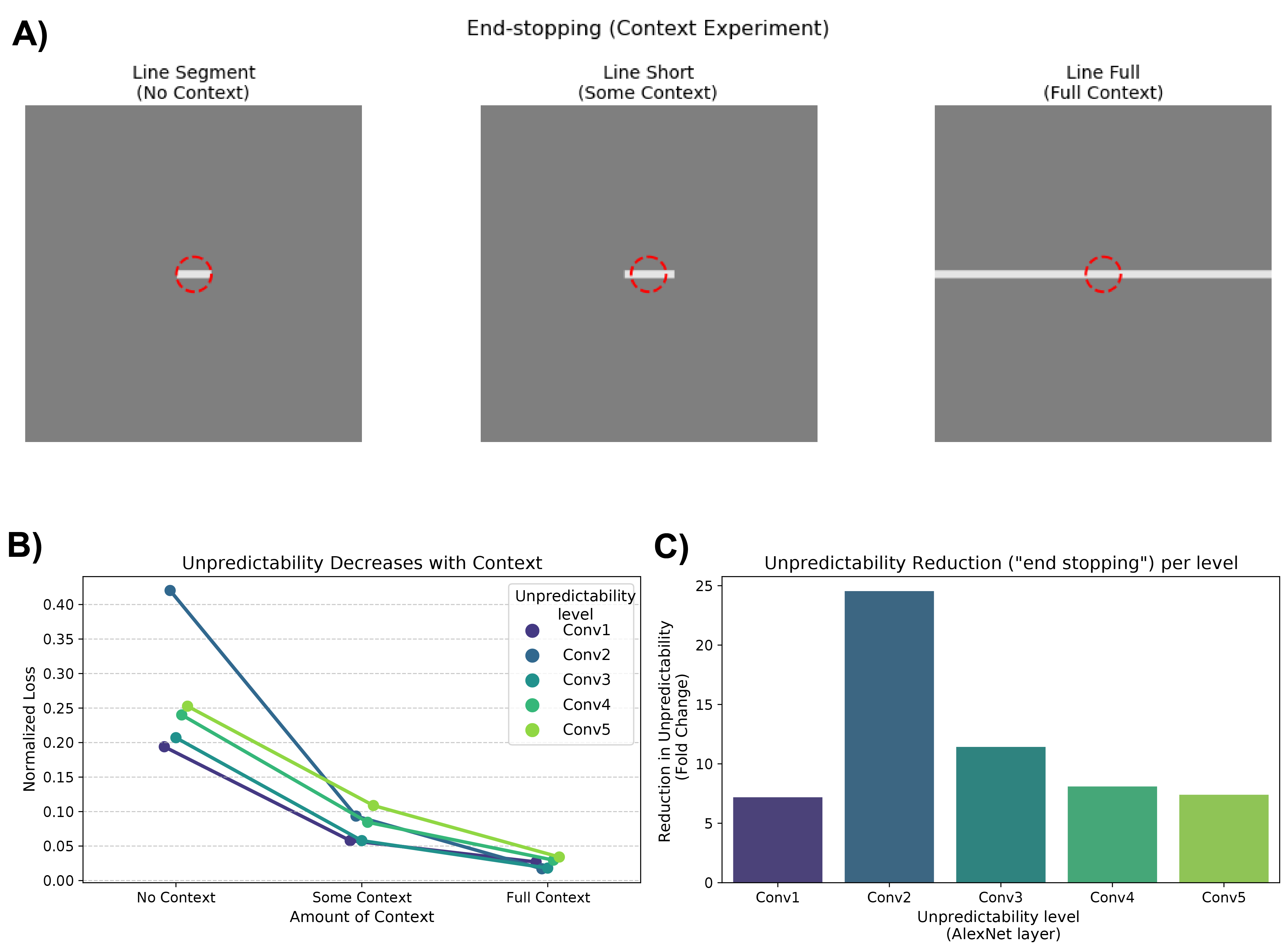

Supplement: S1 Fig — To confirm that our predictability metric is sensitive to canonical forms of spatial context, we simulated a classical end-stopping experiment. A) Three synthetic stimuli were generated where a central receptive field (RF) patch (red dashed circle, corresponding to the in-painted area) was presented with a horizontal line segment. The line was either confined to the RF patch (‘No Context’), extended slightly into the surround (‘Some Context’), or extended far into the surround (‘Full Context’). B) We computed multi-level unpredictability for the RF patch, quantified as the normalized content loss between the actual and predicted patch features from the first five convolutional layers of AlexNet. Unpredictability systematically decreased as more context was provided, consistent with the suppressive effect observed in end-stopped neurons. C) The magnitude of this contextual modulation, computed as the fold-change reduction in unpredictability from the ‘No Context’ to the ‘Full Context’ condition. The effect was feature-specific and strongest for low-level features (Conv2), providing a proof-of-concept that the inpainting-based modelling analysis, designed for natural stimuli, captures fundamental principles of contextual processing with simple stimuli too. (TIFF) [file pcbi.1013136.s001.tif]

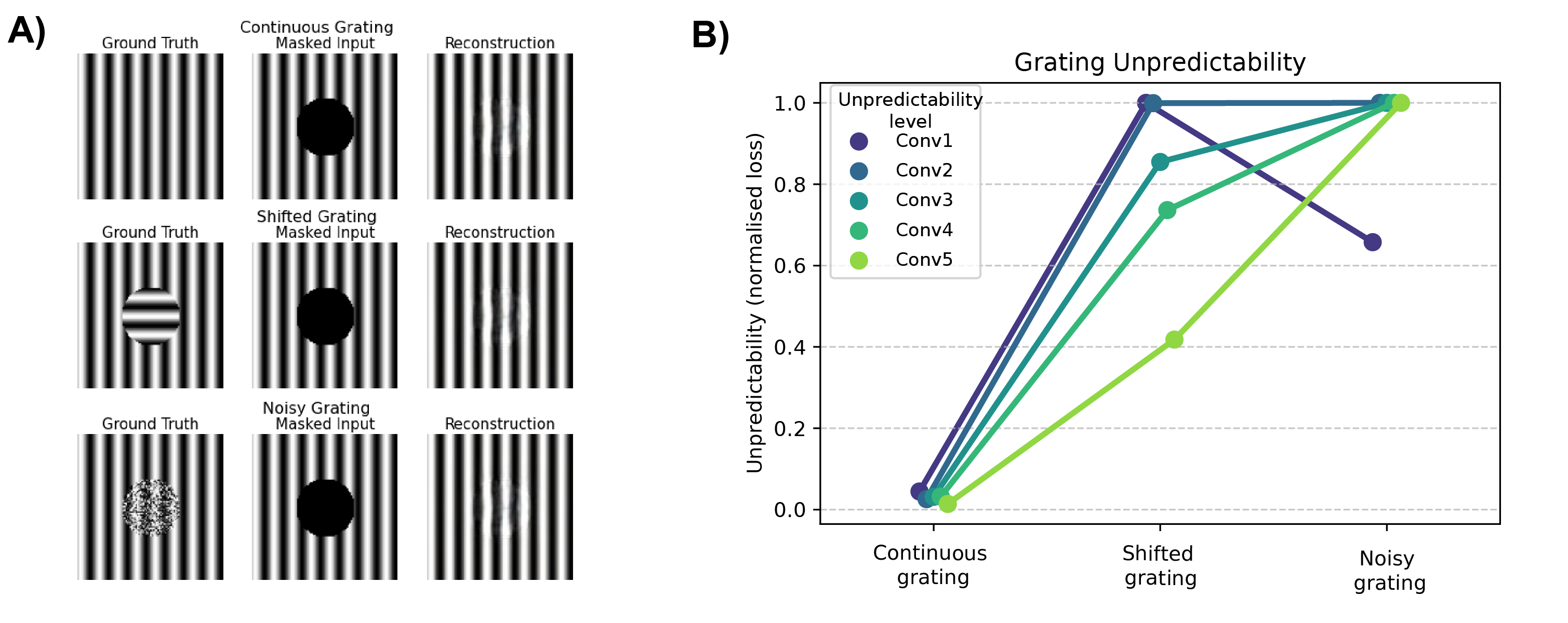

Supplement: S2 Fig — This analysis demonstrates how the multi-level unpredictability metric is sensitive to different feature violations (see Methods). A) We created three ground-truth stimuli where the surround was a continuous vertical grating. The central masked patch contained either a continuous grating (no mismatch), an orthogonal grating (‘Shifted’), or a grating with phase noise (‘Noisy’). Because the masked input to the inpainting model was identical in all cases, the model produced the same reconstruction: a continuous vertical grating. B) Unpredictability was quantified as the normalised content loss between each ground truth and the single reconstruction. This revealed a clear dissociation: low-level unpredictability (Conv1) was maximally driven by the orientation mismatch in the ‘Shifted’ condition. In contrast, high-level unpredictability (Conv5) was much less sensitive to the orientation shift but strongly driven by the textural disruption in the ‘Noisy’ condition. Between Conv1 and Conv5 we observe a gradual shift from being most sensitive to orientation disruption (shifted grating) towards most sensitive to textural disruption (phase noise). This confirms that the metric can distinguish between violations of low-level and higher-level image properties. (TIFF) [file pcbi.1013136.s002.tif]

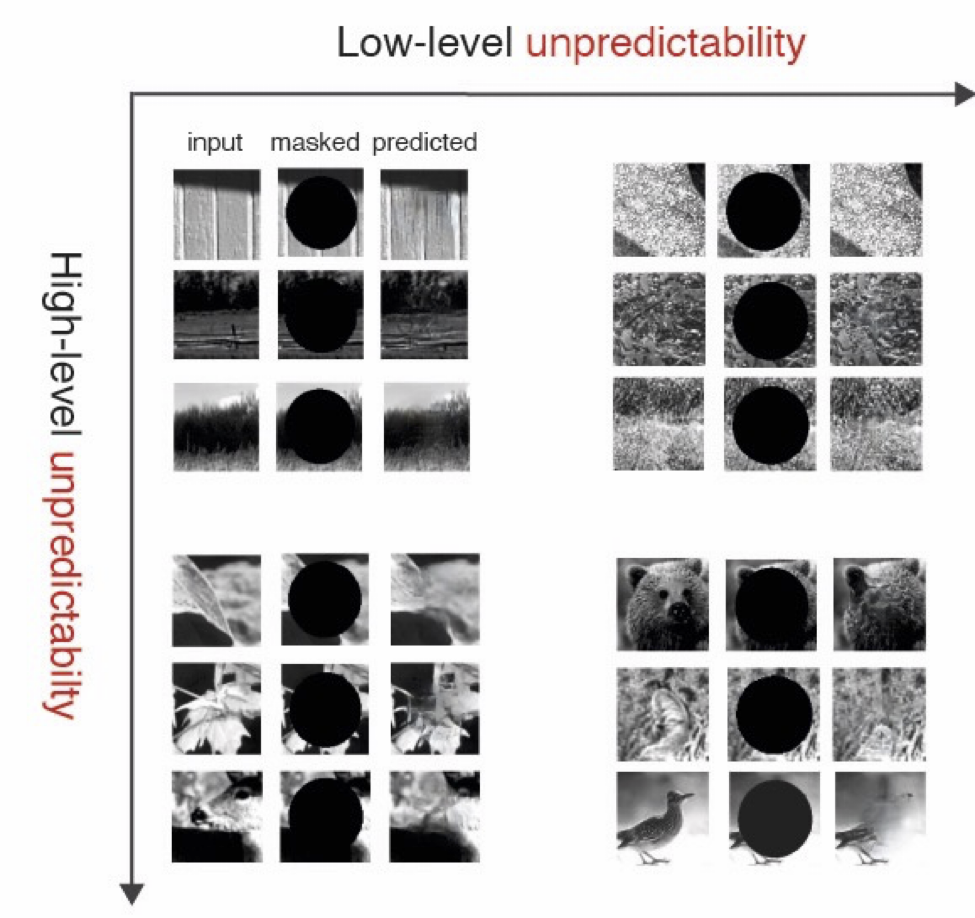

Supplement: S3 Fig — For each of the three examples per quadrant, the figure shows the input patch, the masked version, and the model’s prediction. The quadrants illustrate The quadrants illustrate intuitive dissociations between low- and high-level unpredictability. For instance, patches with low unpredictability on both axes often contain simple, predictable structures like edges, while patches with high low-level but low high-level unpredictability are typically textures. Conversely, high unpredictability at both levels is often observed for complex objects fully spanned by the receptive field. The rarer case of low low-level but high high-level unpredictability can occur for complex objects that are primarily composed of sharp, predictable edges, or other complex objects that contain simple edges. See Methods for more information. All natural images from [1,6]. (TIFF) [file pcbi.1013136.s003.tif]

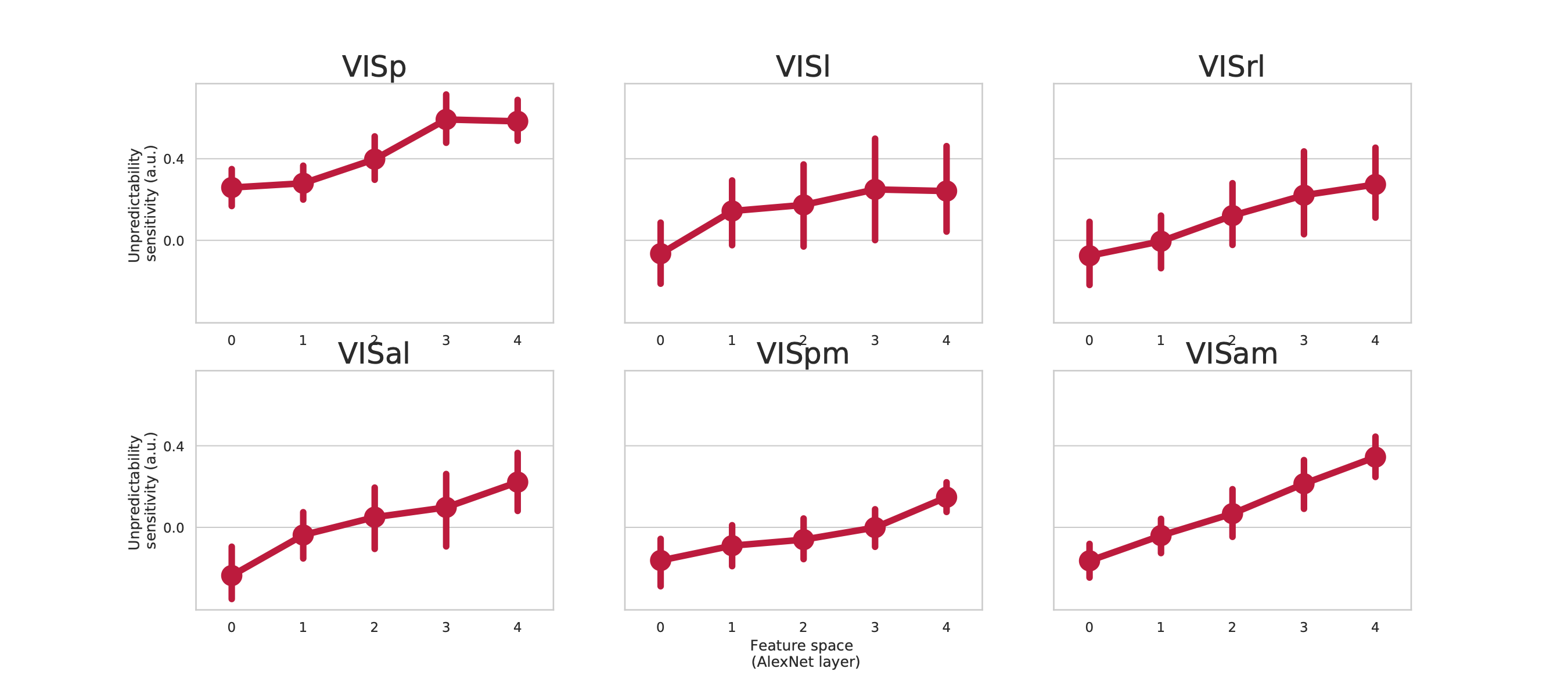

Supplement: S4 Fig — Same as red line in Fig 3 but for all cortical areas. Interestingly, all visual areas show a negative relationship, where sensitivity is highest to the lower-level features. However, in hierarchically higher cortical areas the relationship is more shallow, with less of a preference for lower level features, and lower encoding performance and more variance across units and animals. Dots show mean across animal-level averages; error bars show bootstrapped 95% confidence intervals around the mean. (TIFF) [file pcbi.1013136.s004.tif]

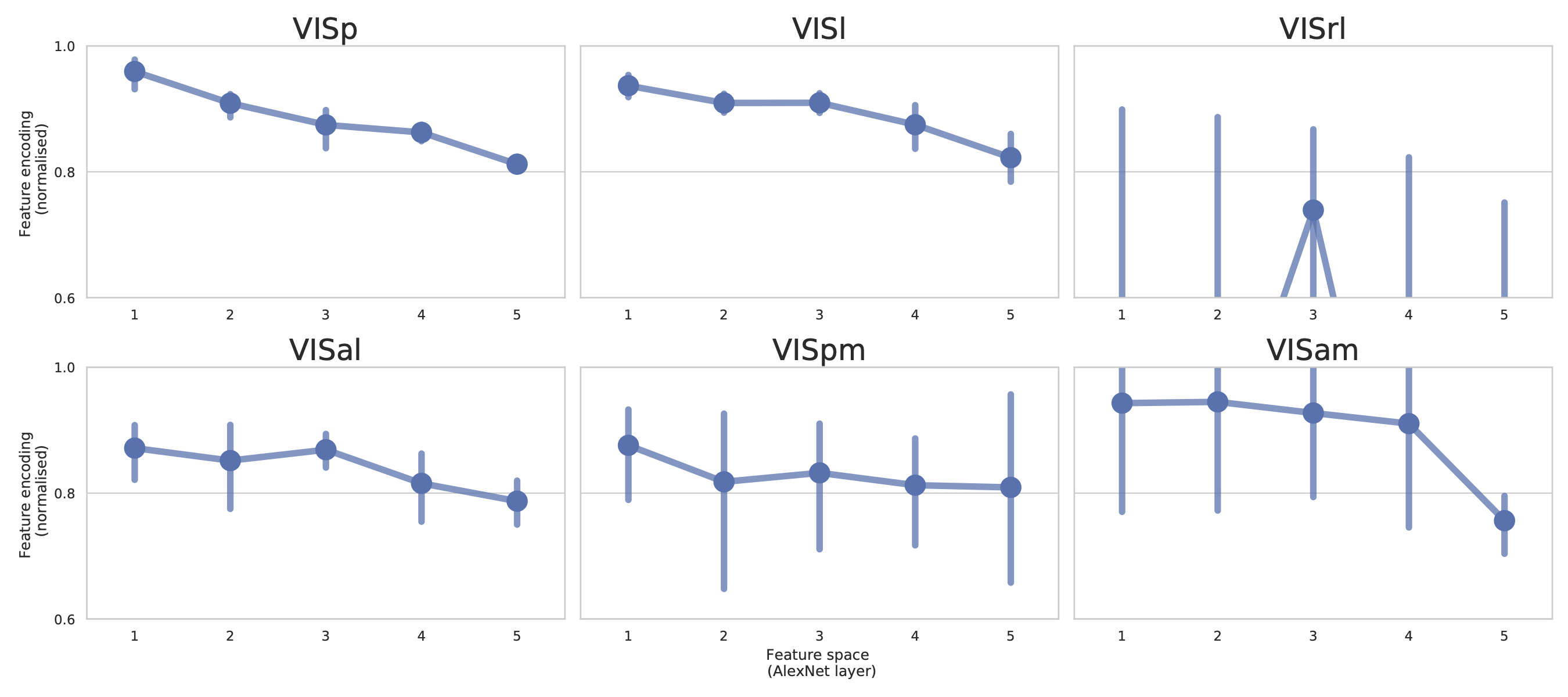

Supplement: S5 Fig — Same as blue line in Fig 3 but for all cortical areas. Interestingly, all visual areas show a similar positive relationship, where sensitivity is highest to the predictability of higher-level features, and lowest for to the predictability of lower-level features. However, in hierarchically higher cortical areas (such as VISam), the relationship appears steeper, so there is a stronger preference for higher-over-lower level features. Dots show mean across animal-level averages; error bars show bootstrapped 95% confidence intervals around the mean. (TIFF) [file pcbi.1013136.s005.tif]

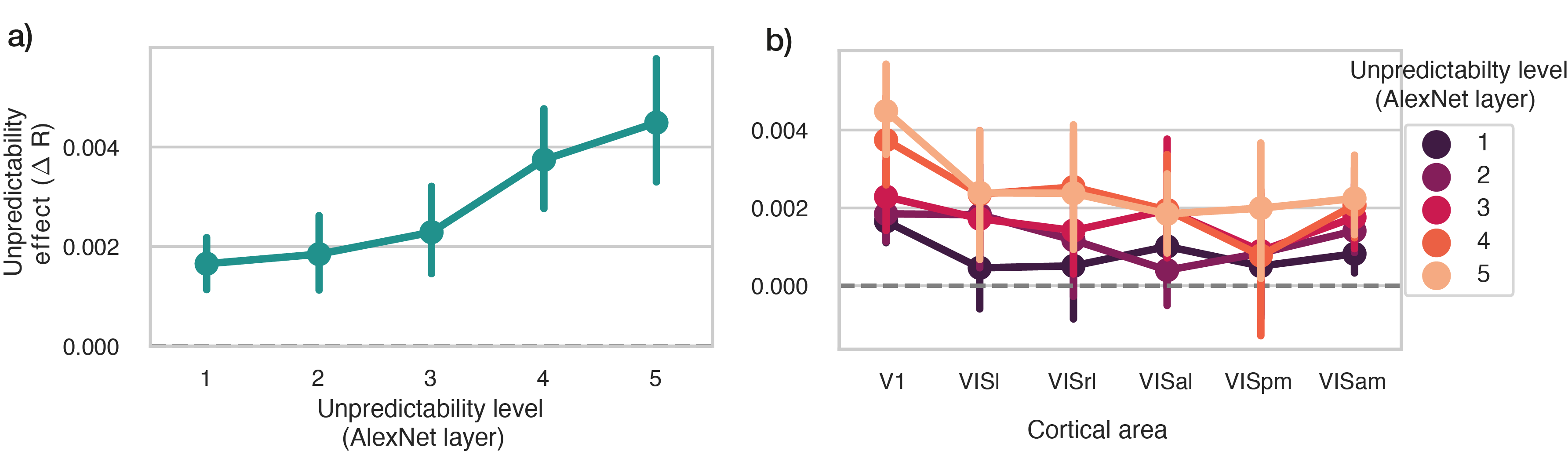

Supplement: S6 Fig — a) V1. Same as red line in 3c, but using the cross-validated ΔR instead of the time-averaged coefficient as a metric of interest. b) ΔR-based unpredictability tuning analysis for all visual areas of interest. X-ticks indicate cortical area, colour indicates feature space or unpredictability level. Just like in the coefficient-based analysis (Figs 3 and S5) we see the same positive relationship in all areas, where the unpredictability sensitivity highest for high-level unpredictability, and lowest for low-level unpredictability. Dots show mean across animal-level averages; error bars show bootstrapped 95% confidence intervals around the mean. (TIFF) [file pcbi.1013136.s006.tif]

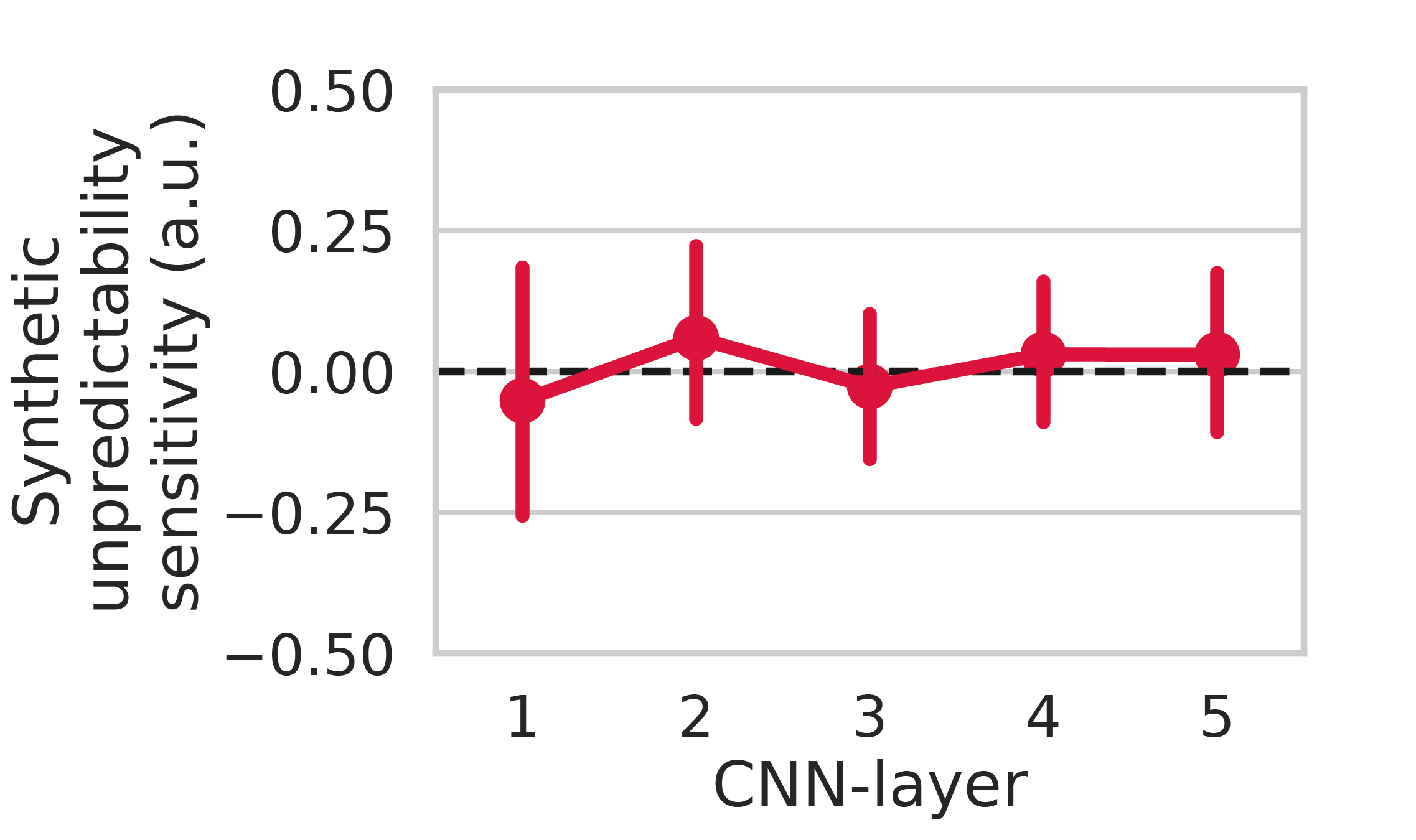

Supplement: S7 Fig — To confirm that the preference for high-level unpredictability (Fig 3c) reflected the neural response to images rather than an inherent property of our method, we repeated the analysis using synthetic noise images. Unpredictability sensitivity is computed as the time-averaged coefficient. Dots show the mean across mice with 95% confidence intervals. No positive trend or any significant effect of unpredictability was observed. (TIFF) [file pcbi.1013136.s007.tif]

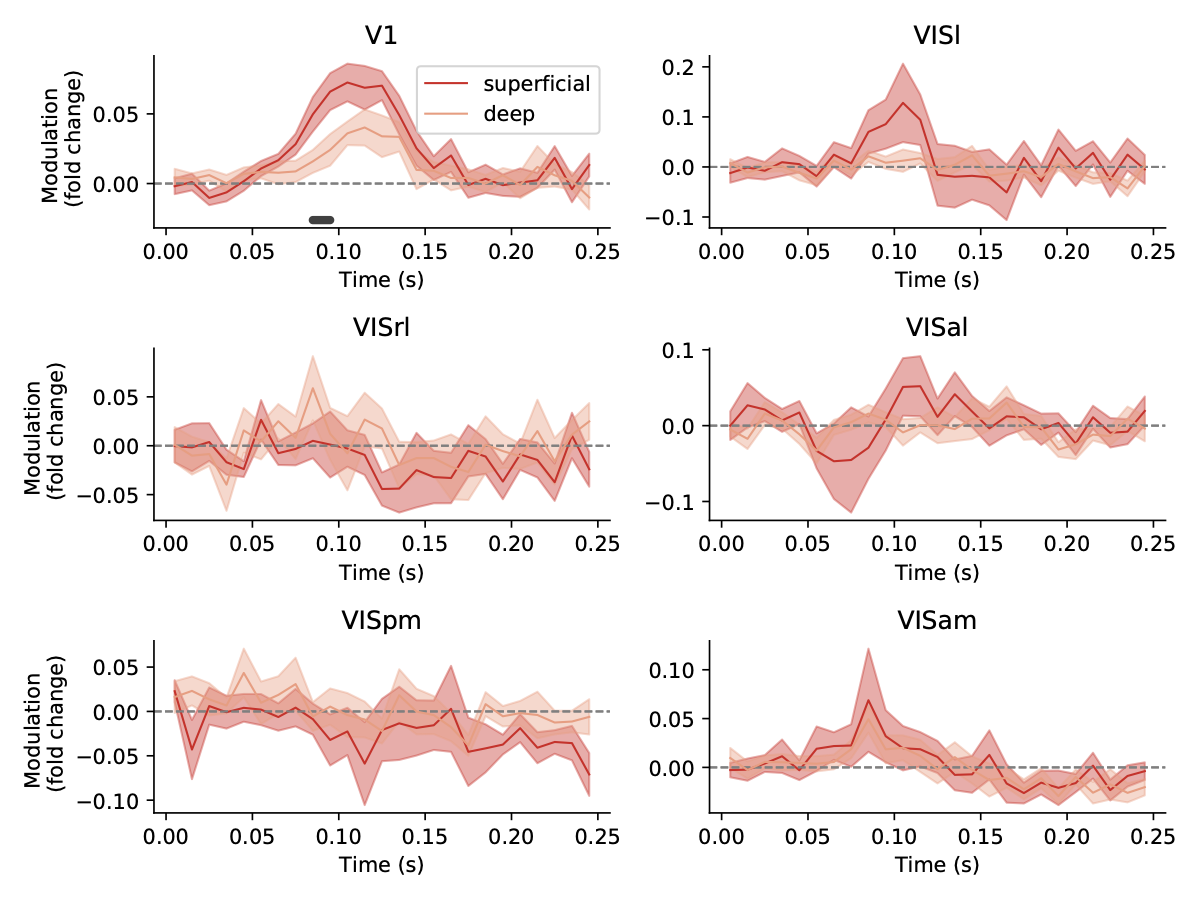

Supplement: S8 Fig — Same as Fig 4d, but for all cortical areas: lines with shaded error bars show the coefficient of overall spatial predictability over time, split out for units classified as ‘superficial’ or ‘deep’. Note that this sub-splitting of already sub-selected units reduces the number of neurons, and the number of animals with enough units in both categories. (TIFF) [file pcbi.1013136.s008.tif]

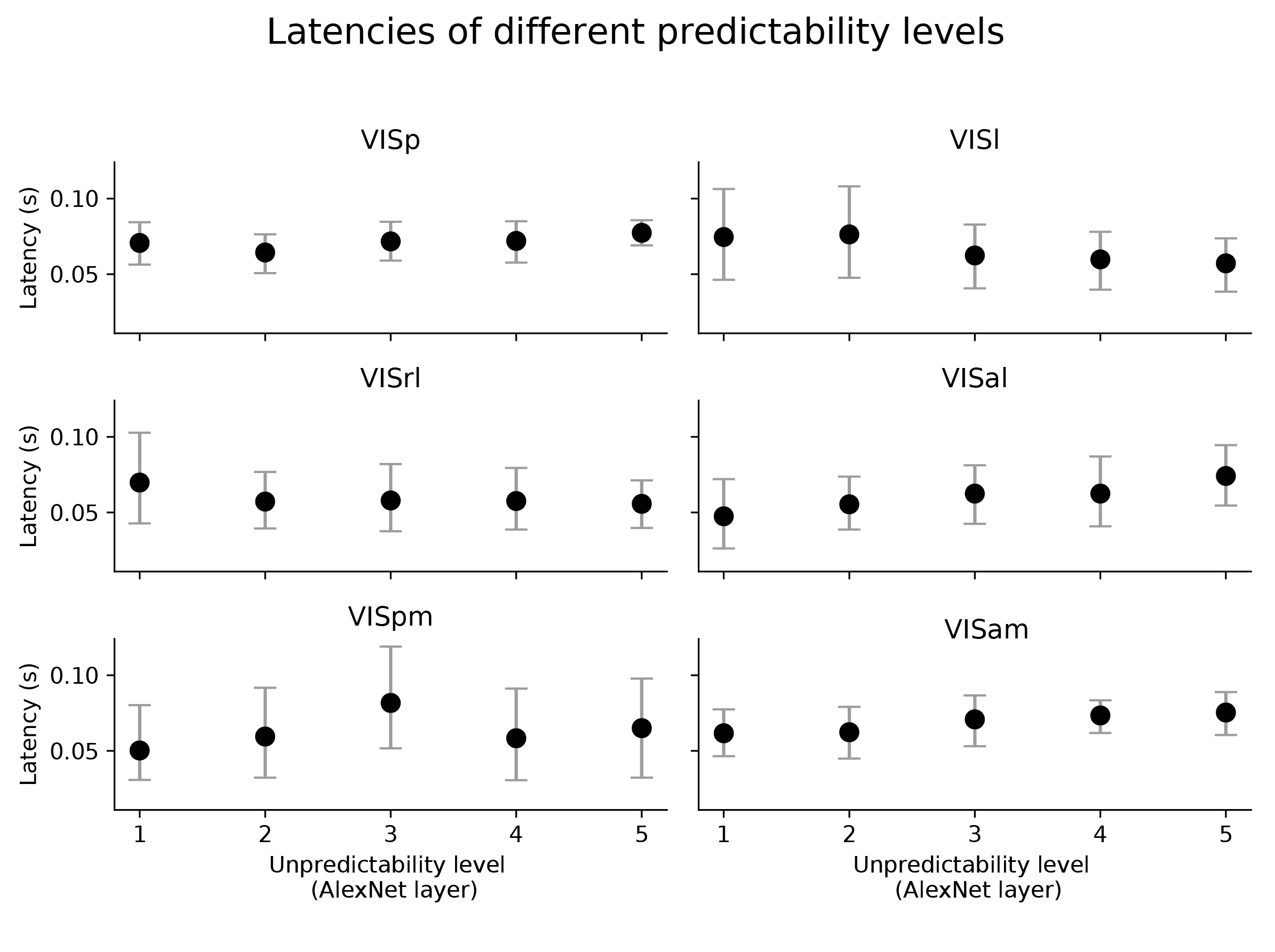

Supplement: S9 Fig — Big dots with error bars indicate mean latency (time to 50% of max), plus bootstrapped 95% confidence intervals across mice. (TIFF) [file pcbi.1013136.s009.tif]
