# Supplementary material for: Higher-level spatial prediction in natural vision across mouse visual cortex
Source: PLoS Comput Biol. 2026 Jan 20;22(1):e1013136. doi: 10.1371/journal.pcbi.1013136 (PMC12829946; doi:10.1371/journal.pcbi.1013136)
Supplement: Attachment — Submitted filename: PCB_reply_to_reviewers_vispred_071125.pdf [file pcbi.1013136.s010.pdf]

Editorial comments:

1) We ask that a manuscript source file is provided at Revision. Please upload your manuscript file as a .doc, .docx, .rtf or .tex. If you are providing a .tex file, please upload it under the item type u2018LaTeX Source Fileu2019 and leave your .pdf version as the item type u2018Manuscriptu2019.

We have reformatted the .tex, and we provide a pdf with tracked changes and without.

2) Please provide an Author Summary. This should appear in your manuscript between the Abstract (if applicable) and the Introduction, and should be 150-200 words long. The aim should be to make your findings accessible to a wide audience that includes both scientists and non-scientists. Sample summaries can be found on our website under Submission Guidelines: https://journals.plos.org/ploscompbiol/s/submission-guidelines#loc-parts-of-a-submission

Thank you, done.

3) Please upload all main figures as separate Figure files in .tif or .eps format. For more information about how to convert and format your figure files please see our guidelines: https://journals.plos.org/ploscompbiol/s/figures

Thank you, done.

4) Some material included in your submission may be copyrighted. According to PLOSu2019s copyright policy, authors who use figures or other material (e.g., graphics, clipart, maps) from another author or copyright holder must demonstrate or obtain permission to publish this material under the Creative Commons Attribution 4.0 International (CC BY 4.0) License used by PLOS journals. Please closely review the details of PLOSu2019s copyright requirements here: PLOS Licenses and Copyright. If you need to request permissions from a copyright holder, you may use PLOS's Copyright Content Permission form.
Please respond directly to this email and provide any known details concerning your material's license terms and permissions required for reuse, even if you have not yet obtained copyright permissions or are unsure of your material's copyright compatibility. Once you have responded and addressed all other outstanding technical requirements, you may resubmit your manuscript within Editorial Manager.
Potential Copyright Issues:
i) Figure 1. Thank you for indicating that “natural images from the Allen Institute Visual Coding Neuropixels dataset.” Please include in the figure legend a direct link to the source of the images and a provide a link to the terms of use / license information.

Images are the raw photographs that were shown to the mice, and specifically the subset of those which are originally the “van Hateren dataset” They are published in the 90’s under a permissive, MIT-like licence by Hans van Hateren, who states that they can be downloaded and distributed freely for scientific, non-commercial uses, and that users are requested to cite the following paper:

van Hateren, J. H., & van der Schaaf, A. (1998). Independent component filters of natural images compared with simple cells in primary visual cortex. *Proceedings of the Royal Society of London. Series B: Biological Sciences, 265*(1394), 359–366.

We have now included this in the manuscript

ii) Please confirm (a) that you are the photographer of 3, or (b) provide written permission from the photographer to publish the photo(s) under our CC BY 4.0 license.

Again, images from Van Hateren, we now indicate this.

ii) Figure 4 . Thank you for stating that it is "adapted from Siegle et al ". Please provide a link to the terms of use / license information.
Note : If the figure is adapted from a copyrighted source, please provide written permission from the copyright holder to publish this under our CC-BY 4.0 license, or remove the figure / replace the image. Please note we do not recommend using standard request forms available on Publishers' websites, as they grant single use rather than republication under an open access license.

Thanks for spotting. The panel was indeed adapted from Siegle et al. We have now removed that specific panel and redrawn a new schematic that conveys the same content.

5) In the online submission form, you indicated that your data will be submitted to a repository upon acceptance. We strongly recommend all authors deposit their data before acceptance, as the process can be lengthy and hold up publication timelines. Please note that, though access restrictions are acceptable now, your entire minimal dataset will need to be made freely accessible if your manuscript is accepted for publication. This policy applies to all data except where public deposition would breach compliance with the protocol approved by your research ethics board. If you are unable to adhere to our open data policy, please kindly revise your statement to explain your reasoning and we will seek the editor's input on an exemption.

We now include a temporary reviewer link, as well as the definitive permalink, which will only become active when the paper is accepted. They both link to the same repository but once the permalink becomes active, the repository is ‘published’ and the entire repository becomes “read-only”.

6) Please amend your detailed Financial Disclosure statement. This is published with the article. It must therefore be completed in full sentences and contain the exact wording you wish to be published.
1) State what role the funders took in the study. If the funders had no role in your study, please state: "The funders had no role in study design, data collection and analysis, decision to publish, or preparation of the manuscript."
2) If any authors received a salary from any of your funders, please state which authors and which funders.

Done

7) Please ensure that grant recipient match between the Financial Disclosure field and the Funding Information tab in the online submission form.

Done

8) Please provide a completed 'Competing Interests' statement, including any COIs declared by your co-authors. If you have no competing interests to declare, please state "The authors have declared that no competing interests exist". Otherwise please declare all competing interests beginning with the statement "I have read the journal's policy and the authors of this manuscript have the following competing interests:"

Done

Reviewers' comments:

Reviewer's Responses to Questions

Reviewer #1: ### **Summary**

This manuscript presents a compelling test of whether spatial predictability of image patches modulates firing rates in mouse visual cortex. By training inpainting U-Nets to reconstruct missing image regions, the authors derive a measure of “unpredictability” within each neuron’s receptive field and include it as an additional regressor in predicting neural responses.

Using this novel method, the authors answered a few very interesting scientific question. Key results are
1) **Unpredictability explains neural variability** beyond classical visual features.
2) **V1 neurons are more sensitive to high-level unpredictability** than to low-level unpredictability.
3) **This effect is slightly stronger in superficial layers** of V1.
4) **Contrast adaptation vs. unpredictability sensitivity**: Stimulus repetitions alter contrast modulation strength (adaptation) but leave unpredictability sensitivity unchanged, suggesting a more stable, long-term role for unpredictability.

Overall, the regression analyses are rigorous and well controlled, making the results—and even the null findings— convincing. The discussion linking higher-level feature prediction to recent advances in self-supervised learning in latent space is insightful.

I recommend acceptance pending minor revisions.

We would like to thank the reviewer for their positive evaluation of our manuscript.

Major Issues

- **Opposing trends in feature predictivity vs. unpredictability modulation**

Figure 3C highlights intriguing, opposite trends between feature predictivity and unpredictability modulation. To ensure these trends are not inherently anticorrelated (e.g., due to differences in scale or variance across feature layers), the authors should consider including a synthetic control. For example, apply the same regression pipeline to random pixel functions or to activations of random neural-network units—this would demonstrate that the observed anticorrelation is not a mere result of the features, but something to do with neuronal tuning..

- One concrete question is: Are regression coefficients directly comparable across different AlexNet layers, or might layer-specific variances drive these differences?

To answer the concrete question: all variables in the regression models are z-scored, so the regression coefficients are standardized effect sizes, and the trends cannot be caused by differences in scale or variance across layer features.

To be absolutely sure the differences we find are reflective of neural tuning and not of some aspect of the computed features themselves, we have re-done the predictability computations but now with synthetic (noise) images, instead of natural images, like the reviewer helpfully suggested.

First we confirm that the pipeline works on non-natural images, which is indeed the case:

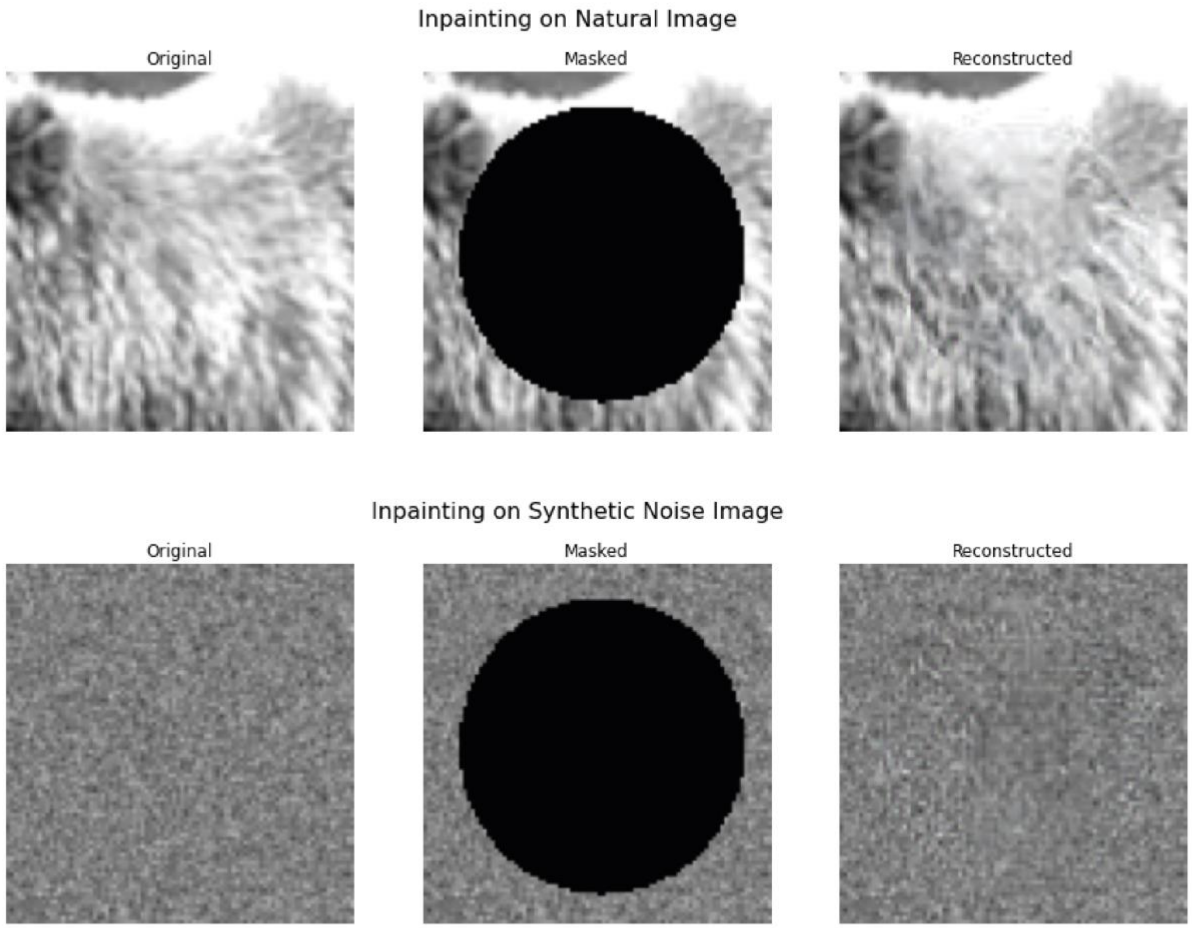

Figure R 1, illustration of spatial prediction (reconstruction) analysis with natural images from the dataset (upper panel) and synthetic noise images (lower panel)

To rule out the that the observed gradient was caused by some inherent structure in the predictability model we redid the entire analysis, assigning a fixed random noise image for every natural image, computing the requested “synthetic predictability” metrics, for every neuron, in every mouse.

We then re-performed the entire regression analysis, but replacing the ‘real’ predictability features with ‘synthetic’ predictability features.

Reassuringly, we did not observe any differences in sensitivity, the preference for more “high-level unpredictability” was not observed:

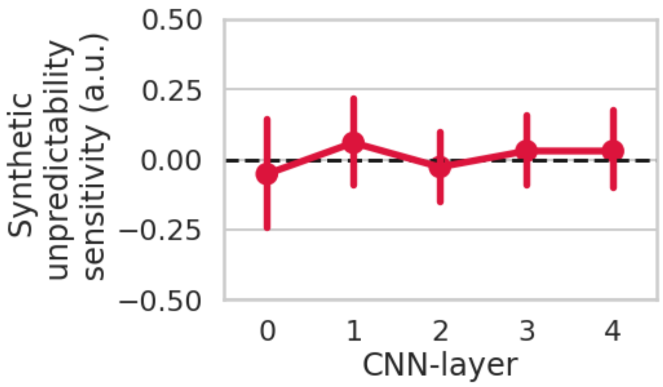

Figure R 2. Unpredictability sensitivity for multiple levels of unpredictability with synthetic noise images as a control. Unpredictability sensitivity is computed as in Figure 3 (time-aggregated average of coefficient), dots with bars indicate mean across mice with bootstrapped 95% confidence interval. No effect nor any preference for higher-level unpredictability.

We have now added this analysis in the supplement.

- **Statistical robustness of the depth–predictivity relationship (Figure 4A)**

The depth–predictivity relationship appears noisy, with several flat or reversed trends. The authors should apply alternative statistical tests to confirm its robustness.

- In terms of plotting style, in Figures 4B–C, large markers occlude underlying data. Consider reducing marker size or adding transparency to reveal individual points and trend lines.
- If the result does not achieve statistical significance, the authors should temper the language in Section 2.3’s title.

We agree the depth predictivity relationship (Figure 4) is not very strong. At the same time, we should stress that the test was based on a pre-defined time-window (75-150 ms) and uses the same procedure of across-timepoints and across-neuron aggregation we use throughout the manuscript.

As an alternative test, we now also performed a comparison in a time-resolved fashion, to rule out it was specific to only this time window. More specifically, we computed the difference (coefficients for CNN-layer-unspecific, overall unpredictability: ‘superficial’ – ‘deep’ neurons), and tested for significance at each timepoint. To correct for multiple comparisons (FWER-correction), we used threshold-free cluster enhancement (TFCE), using default parameters from MNE python, which returns a FWE-corrected p-value at each timepoint. This shows significant differences between 85 and 110 ms:

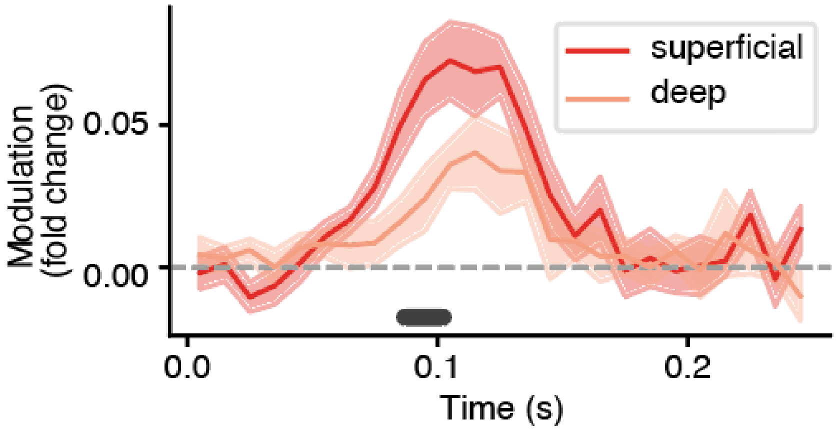

This in turn confirms that the small but significant effect in the pre-defined time window (75-150ms, used for all analyses throughout the paper) was not a “lucky shot” unique to this particular time window.

To maintain consistency with the other statistical tests throughout the paper, we still primarily focus on the pre-defined time window and only add the temporally resolved analysis as a complementary analysis, and in the supplementary material, in the Figure of all ROIs:

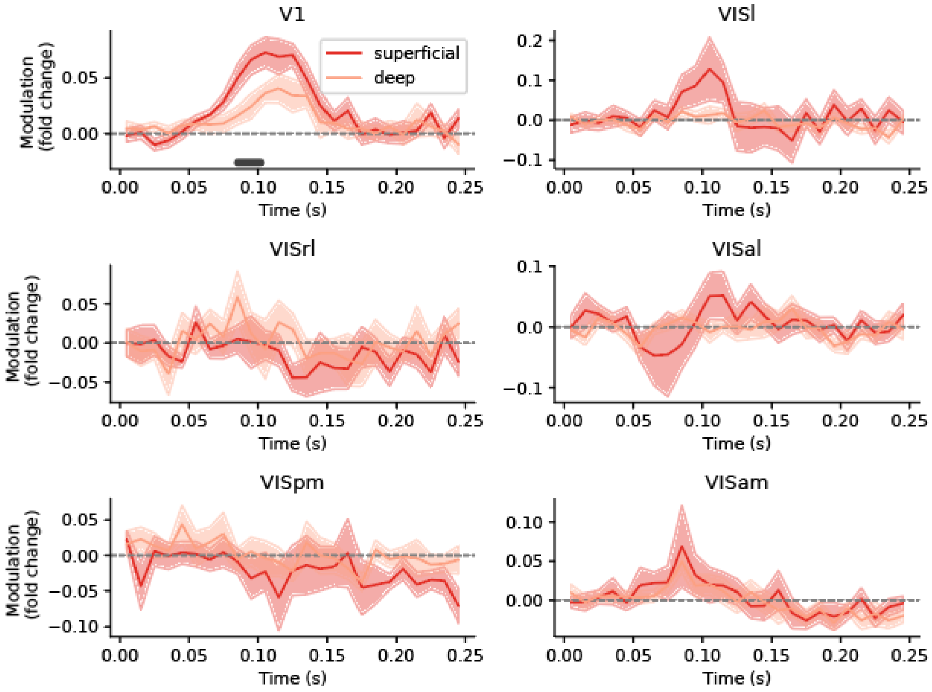

- **Use of** ΔR^2 **to quantify unique contributions**

The manuscript reports changes in correlation (ΔR), but correlation coefficients are not really additive. A more rigorous metric is the change in explained variance (ΔR^2), which

directly reflects each variable's unique contribution. Please compute and report ΔR^2 to verify consistency with the ΔR findings. I guess most of them should be the same.

We appreciate the reviewer's comment regarding ΔR^2. We agree that R^2 is the appropriate metric for quantifying the unique variance explained by a variable.

However, our primary goal was to test for a robust increase in cross-validated prediction performance, rather than to quantify the amount of unique explained variance. **For the purpose of demonstrating a consistent increase in prediction performance, ΔR is a suitable metric.** To ensure the robustness of our findings, we repeated the analyses using ΔR^2 -- defined as the coefficient of determination.

We can confirm that the conclusions remain roughly unchanged:

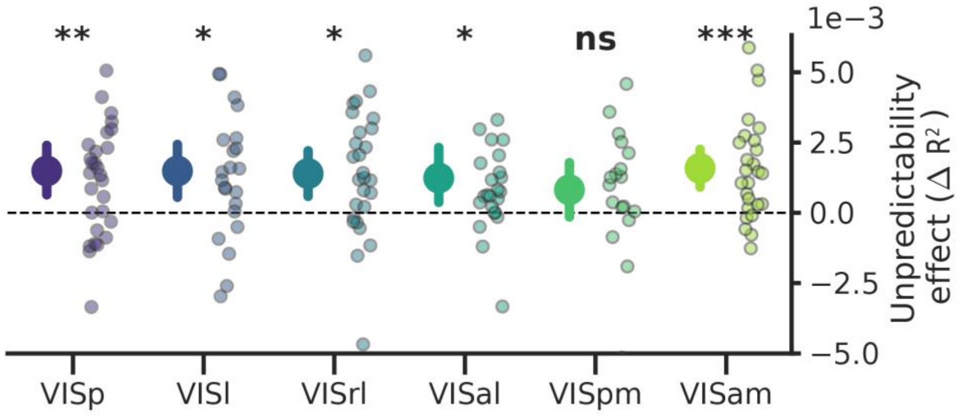

*Figure R 3. Like Figure 2b, but using R2 instead of R. Specifically, increase in cross-validated performance, quantified with coefficient of determination, between 75-150 ms when adding overall unpredictability to the baseline model. Small dots indicate individual animals; large dots with error bar indicate mean across animals and bootstrapped 95% CI. Stars indicate statistical significance, after Bonferroni correction: * P< 0.05; ** P<0.01; *** P < 0.001.*

However, we would prefer to keep using R and ΔR, rather than R^2 and ΔR^2. This is because, when using cross-validation – and when defining R^2 via the residuals, so as 1 minus the ratio of the residual and total sum of squares – R^2 can become **arbitrarily negative** for noisy neurons, which can introduce skewed outliers. **This can increase the variance in the results**. To illustrate this, we recreated Figure 2a, the time-resolved cross-validated prediction performance of the baseline model across the visual cortical areas, now using R^2:

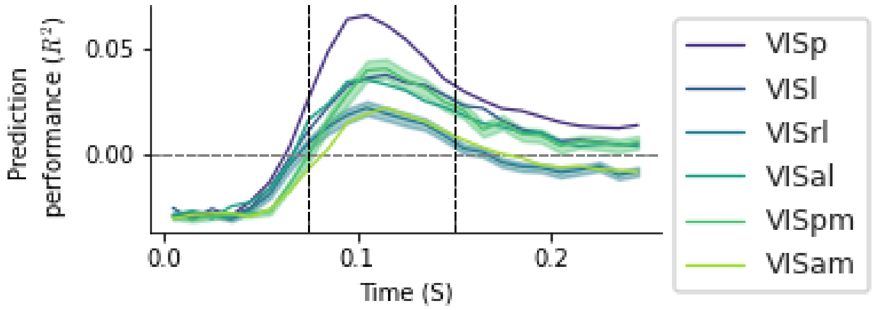

Figure R 4, Time-resolved cross-validated prediction performance of the baseline model across the visual cortical areas. Same as Figure 2a, but using R2, i.e 1 minus the ratio of residual sum of squares and total sum of squares.

If we compare this to the original plot with cross-validated R:

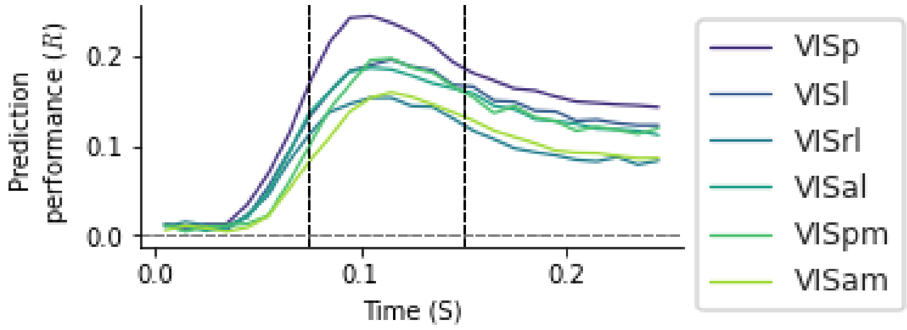

Figure R 5. Same as Figure 2a of paper.

It is clear that when using R^2 the results are more noisy and track the established hierarchy between the visual areas (Siegle et al. 2021) less clearly.

(Note that in Figure R4 R^2 is negative until about 70 ms, indicating higher resisual error than the total sum of squares, i.e. a null model predicting only mean. Moreover at multiple timepoints the R^2 is lower than square of the cross-validated correlation coefficient (sometimes called r^2). This points to scaling issues, e.g. due to unexpectedly silent or active neurons in the test split, rather than only unexplained variance.)

Altogether, we prefer R as a metric, but have updated the results and code for the confirmatory check with coefficient of determination to the codebase.

- **Interpretation of the Split-Half Analysis Results**

We suggest refining the title and framing of Section 2.4 to emphasize that repetition-driven adaptation is more parsimoniously explained by modulations in contrast sensitivity than by changes in unpredictivity.

Unpredictivity, as computed here, depends solely on the stimulus image and the neuronal

receptive field. Just like classical image features (e.g., contrast, spatial frequency), unpredictivity remains invariant across repeated presentations of the *same* image. Consequently, any repetition-related change in the regression weights must reflect an adjustment in the *relative* strength of these features. So the analysis of using the same unpredictivity measure to regress first and 2nd split of data seems to be misaligned with the goal of examining short term effect on predictivity modulation. Could it be that the predictivity metric itself changed in the first and 2nd half due to exposure?
Therefore, we recommend tempering the language in the current Section 2.4 title (and corresponding conclusions). The authors could clarify this in their response, it's possibly my confusion.

Perhaps we have been unclear. To be sure, we did not aim to claim that adaptation was *driven by* changes in unpredictability.

Instead, we asked whether the unpredictability effect was itself affected by repetition: i.e. whether the modulation by *the same degree of unpredictability* (which is indeed invariant across repetitions) would be affected, e.g. strengthened after increased exposure to the stimulus. In other words, it could be that, over increased familiarity with the stimulus, visual cortex is increasingly able to exploit the spatial structure (predictability) in the images, and hence later trials would exhibit a *larger* difference between high hand low predictability stimuli / image patches than early trials.

It is true that this analysis can only be valid if there are no sizable differences in the different halves. I.e. if the first half contains many more "high predictability" stimuli than the second half. However, because the way the stimuli are presented, this is not the case. To confirm this, we provide a histogram of image identity in the first and second halves of trials, and the average unpredictabilities below.

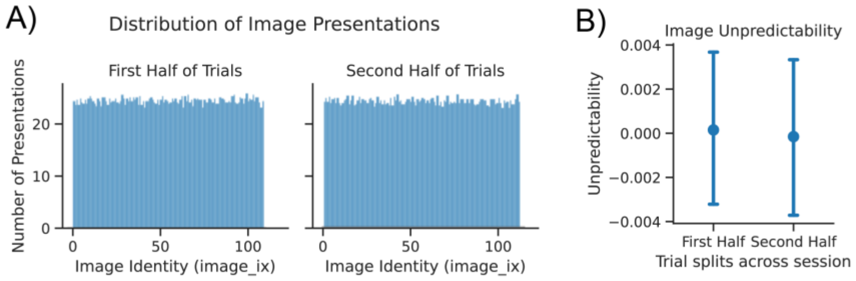

Figure R 6. A) Histogram showing number of presentations of each image in first and second half of trials. B) Average global (cnn-layer-unspecific) unpredictability, for first and second half. Dots indicate mean, error bars indicate bootstrapped confidence interval around the mean.

Minor Concerns

- Captions for Figures S1 and S2 appear mismatched or contain typos; please correct them. As a side note, I also found the trend across visual hierarchy quite interesting, authors could consider move some of them to Figure 3.

Thanks, fixed.

- In the Methods, authors can include explicit regression equations to improve clarity.

We have now included equations, as requested.

- For Figure 2, clarify whether inputs (contrast energy, unpredictability) were normalized (e.g., z-scored) before regression; without normalization, beta coefficients cannot be interpreted as the modulation strength as the authors did.

All predictor variables are z-scored, as outlined in the methods. We now add it to the caption for improved clarity.

Reviewer #2:

This work develops a computational method that investigate the neural mechanisms of predictive vision in mice, following up on earlier primate work. Using the Allen Visual Coding Dataset, the authors demonstrate that predictive processing principles seen in macaque V1 also hold in mouse VISp, including higher-order visual areas, in laminar resolution. The methods are clearly explained and the analyses and controls are in place. My suggestions are minor—mainly simplifying some wording to improve readability and, if possible, visualizing a few extra insights already covered in the results / discussion sections.
Overall, I highly recommend the manuscript for publication.

We would like to thank the reviewer for their positive assessment of our manuscript.

I have one major suggestion, which is to simplify the wording around “predictability”. Predictive coding terminology is already quite loaded and “predictability” tends to mean different things to different people, like spatial or temporal predictability. It would be very useful if the definition is clarified at the beginning of the results section (or introduction), so the reader won’t rely on priors (and the rest of the text follows that definition of predictability). I find that “predictability, unpredictability, multi-level predictability, low/high-level predictability” are used in place and clear. However, there are multiple phrases that refer to the same definition of “predictability”:

Predictability, spatial predictability, visual spatial predictability, stimulus predictability, image predictability, image patch predictability, image feature predictability - and they are sometimes used one after the other, which likely confuses the reader. Some instances are:

Section 1 introduces spatial/stimulus predictability,
Figure 1 caption use both spatial predictability, image patch predictability,
Results Section 2.1 highlight image patch predictability, used in section 2 text, Figure 4, Supp. Figure captions
Section 2.2 title: Image predictability

Methods sections 4.3 and 4.5 use Spatial predictability
Similarly for high-level predictability: high(er)-level feature predictability

Overall, I find simplifying some of these variations would improve readability for the reader and improve the manuscript.

We fully agree with the reviewer.

We have standardized our terminology to consistently use **"**spatial predictability**"** throughout the manuscript (replacing "image patch predictability", "image predictability", etc.).

We retained two minor exceptions: (1) "stimulus predictability" appears in the Introduction when referring to the broader predictive processing literature, as it encompasses both spatial and temporal predictions; (2) we use "higher-level" rather than "high-level" as a relative term that accurately reflects our graded feature hierarchy without overstating the semantic nature or "high-levelness" of features in mouse visual cortex and later convolutional layers in AlexNet.

These revisions substantially improve readability while maintaining conceptual precision.=

I have a few minor comments:

Figure 1 seems a little crowded, (all) methods could be less described - I don't have a clear suggestion.

We wanted the figure to convey:

1) The experimental paradigm (passive viewing, 250 ms, of complex natural images); panel **A**)
2) Analyses are done for individual neurons, for every receptive field, separately, selecting RFs **(B)**
3) The general logic (of the analysis; panel **C).**
4) The fact that we use extensive baseline variables; panel **D).**

We have considered but could not come up with simpler way to convey all these aspects.

Figure 3.b) example images are helpful and intuitive for the concepts of high vs. low level predictability, the authors might consider including more example images if there is room for it.

We are glad the examples images are helpful. We have now added more examples, 3 per quadrant. We have added these as a figure in the supplement, because it didn't fit in the main figure:

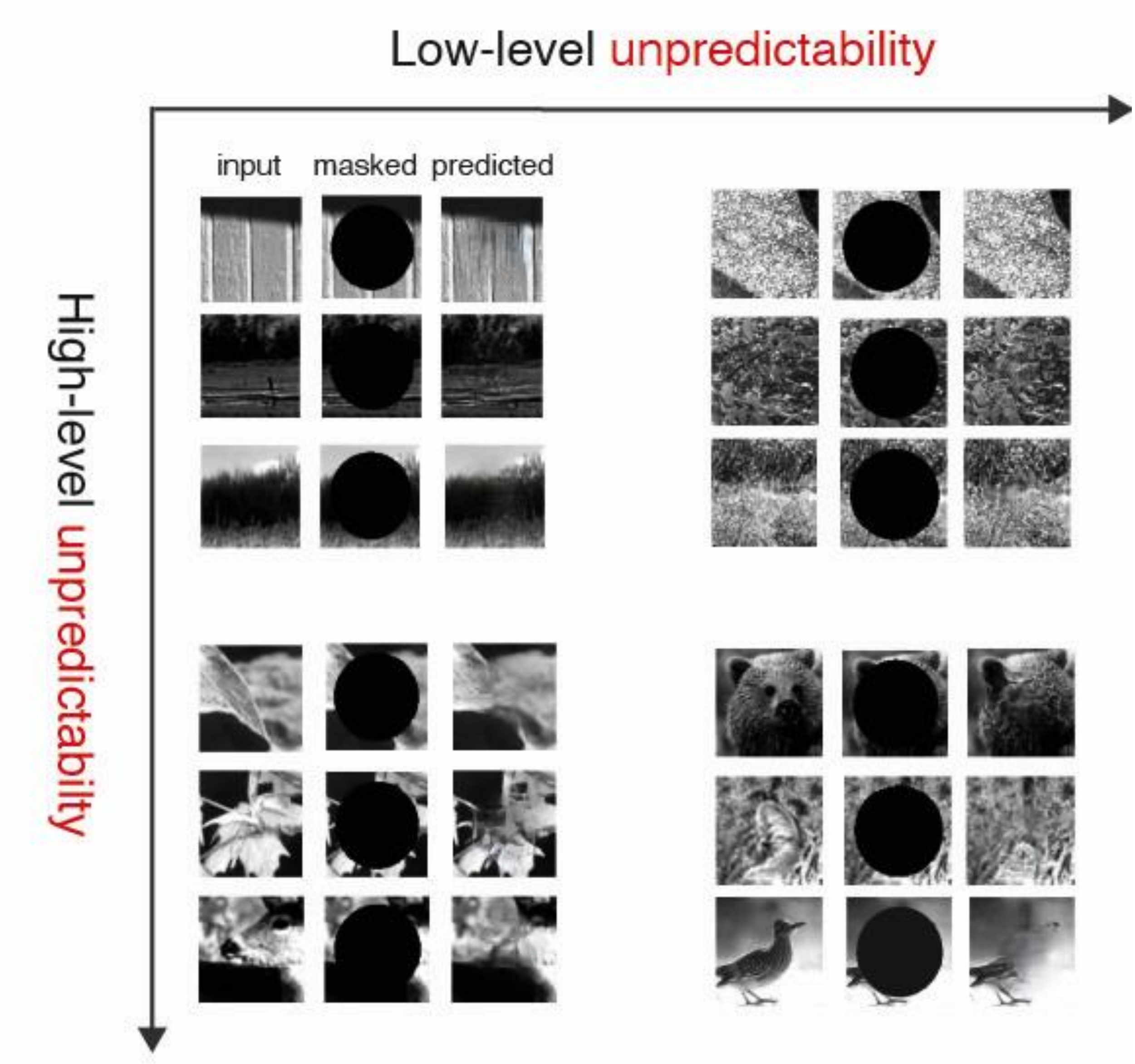

Figure R7 ***Example patches for each of the four quadrants of feature-level predictability estimates.*** *For each of the three examples per quadrant, the figure shows the input patch, the masked version, and the model's prediction. The quadrants illustrate The quadrants illustrate intuitive dissociations between low- and high-level unpredictability. For instance, patches with low unpredictability on both axes often contain simple, predictable structures like edges, while patches with high low-level but low high-level unpredictability are typically textures. Conversely, high unpredictability at both levels is often observed for complex objects fully spanned by the receptive field. The rarer case of low low-level but high high-level unpredictability can occur for complex objects that are primarily composed of sharp, predictable edges, or other complex objects that contain simple edges. See Methods for more information.*

Typo on page 3 of manuscript: indepdent
Typo on page 5 of manuscript, Fig. 3 caption: unpredictabillity
Typo on page 9 of manuscript: chacterised
Typo on page 19 of manuscript: Figure S2 caption: feature

Thanks, fixed.

Reviewer #3:

This manuscript proposes to test predictive coding in data from mouse visual cortex. The authors use publicly available recordings from Allen Institute, including thousands of neurons from multiple visual cortical areas, while animals passively view static natural images flashed briefly on the screen. They use deep generative model based on the UNet architecture to quantify spatial predictability, namely how well a masked portion of the image can be reconstructed from the rest of the image (the surround or spatial context). They then use a linear regression approach to quantify the sensitivity of neurons to image predictability, namely the improvement of a linear fit to the firing rate across multiple images, when adding predictability as an independent variable in addition to low-level image features (e.g. contrast) and non-visual variables like running speed. The main finding is modulation by unpredictability, i.e. higher firing rate for unpredictable images, suppressed firing rate for predictable images. Another interesting finding is that the modulation is stronger when predictability is defined by deep feature layers (presumably more closely related to perception) than low-level features. Conversely, the base encoding model relies more on low-level features. This is interpreted as consistent with the idea that predictions are computed recurrently and via feedback, while feedforward processing encode low-level features.

Overall, the approach is interesting but somewhat underdeveloped, with some important choices not motivated; the theory is only outlined in words so some of the predictions (e.g. about layer specificity) are difficult to interpret in the data; and the main effects in the data are small to the point that it is unclear how meaningful they are. Some key references are missing and/or not sufficiently discussed. The manuscript is written clearly although some technical details could be added. Details are provided below.

We would like to thank the reviewer for their assessment of our manuscript.

1. Definition and quantification of predictability
This is the key metric, but not much is done to establish that it is a valid metric. It is not obvious that it is a rigorous perceptual predictability metric, and even less so that it is meaningful for visual cortex neurons. For the latter, one important control would be to apply it to gratings, bars, textures, noise stimuli etc. with which contextual effects have been studied in V1, and test if predictability so defined reproduces classical effects. If it does, is there something unique about this metric, or would any other intuitively meaningful metric of predictability/redundancy work as well? If it does not, how is one to interpret the results, i.e. that cortex is sensitive to predictability for natural images but not simpler parametric stimuli?

We thank the reviewer for raising these points. We agree that clarification is warranted and have addressed this in two main ways: 1) by refining the manuscript's text and 2) by performing two new analyses on simple, parametric stimuli, which we have added to the supplement.

1) Clarifying the unpredictability metric

As to whether the metric is valid or meaningful, we should clarify that this metric is well-established in computer vision, where multi-level unpredictability (content loss) is widely used to train in-painting models (alongside other loss functions such as style loss). Beyond the domain of in-painting, deep feature representations are known to be highly effective for quantifying perceptual similarity between images or image patches (Zhang et al., 2018). Moreover, we build on Uran et al. (2022) who pioneered its use in neuroscience, and performed comparisons with other spatial predictability metrics. The advantage of this DNN-based approach is its ability to quantify predictability in the rich feature spaces of natural stimuli, which cannot be easily parameterized. We now clarify this in the methods (new text in red):

> *...thus providing a more robust measure of perceptual distance and hence predictability (Zhang et al., 2018).*
>
> *Fundamentally, our metric provides a quantitative measure of a stimulus property: the difficulty of spatially predicting a local patch from its context. This approach is a direct application of content loss, a common objective function used to train generative in-painting models, first used in neuroscience by Uran et al. (2022). The critical advantage of this DNN-based approach is its ability to provide a continuous, quantitative measure of predictability for naturalistic stimuli, across rich, open-ended feature spaces that cannot be easily parameterized.*
>
> *In our analyses, we use two types of spatial predictability estimates...*

2. Validation on synthetic stimuli:

The primary strength and goal of our approach is its application to natural images. Indeed, the in-painting model was exclusively trained on natural images. Simple, parametric stimuli are therefore 'out-of-distribution' by design. Nonetheless, we appreciated the suggestion to bridge to more classic neuroscience findings. We therefore performed two new analyses to check whether this approach can qualitatively capture spatial predictability effects using simple parametric stimuli.

In our first new analysis (Fig. R8, Figure …), we qualitatively simulated an "end-stopping" experiment, in which the spatial unpredictability (prediction error) was measured as a function of the degree of extension of a horizontal bar beyond the inpainting mask (“receptive field”) We found that, as a line segment extended further into the spatial context ("Full Context"), the unpredictability of the central patch decreased. And the decrease (which can be seen as analogous to a decrease in firing in an end-stopping neuron) was strongest for low-level unpredictability (conv2). This result qualitatively recapitulates the suppressive effects of “hypercomplex” end-stopped neurons in V2, providing a proof-of-concept that our metric is sensitive to this classic forms of spatial context:

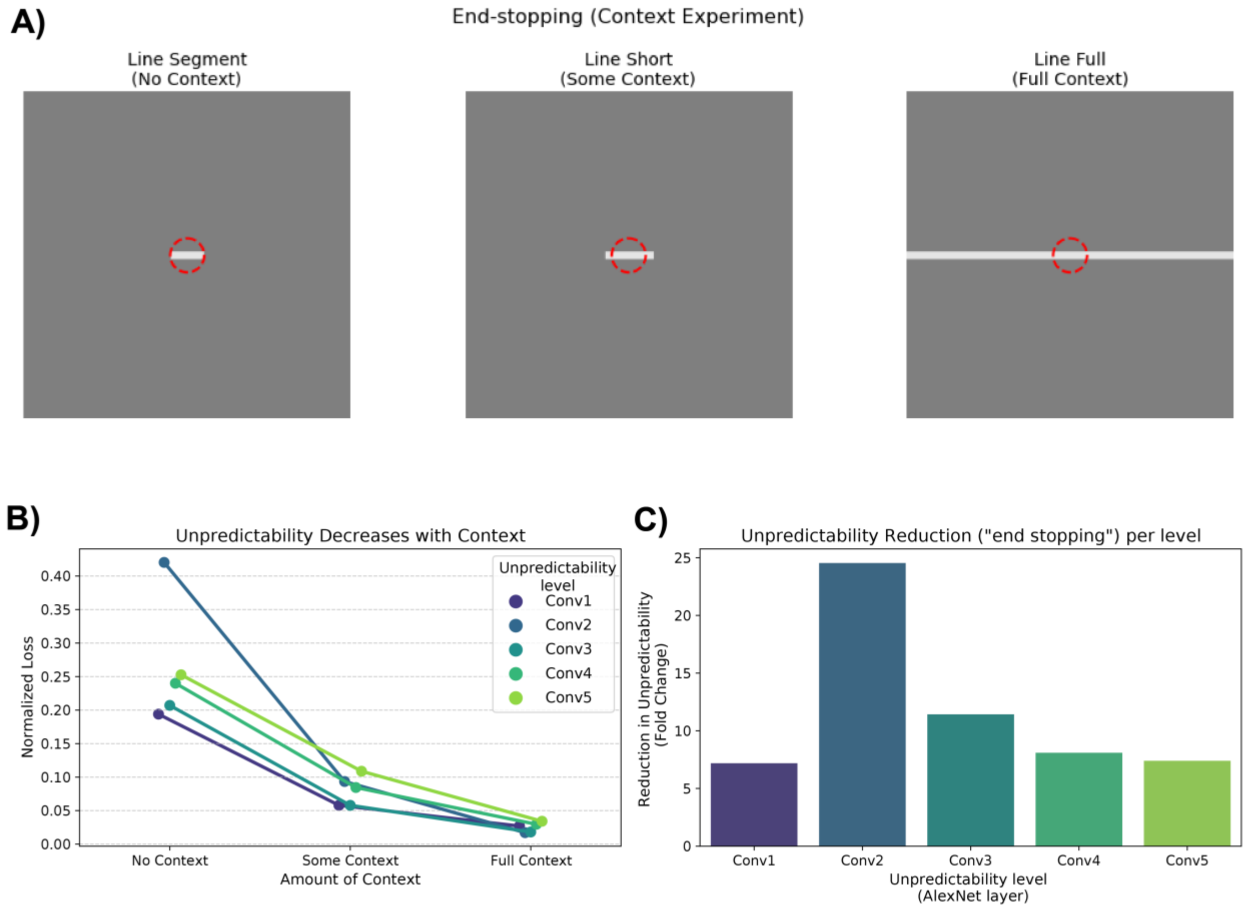

***Figure R 8 Inpainting-based unpredictability qualitatively recapitulates classical end-stopping**. To confirm that our predictability metric is sensitive to canonical forms of spatial context, we simulated a classical end-stopping experiment. A) Three synthetic stimuli were generated where a central receptive field (RF) patch (red dashed circle, corresponding to the in-painted area) was presented with a horizontal line segment. The line was either confined to the RF patch ('No Context'), extended slightly into the surround ('Some Context'), or extended far into the surround ('Full Context'). B) We computed multi-level unpredictability for the RF patch, quantified as the normalized content loss between the actual and predicted patch features from the first five convolutional layers of AlexNet. Unpredictability systematically decreased as more context was provided, consistent with the suppressive effect observed in end-stopped neurons. C) The magnitude of this contextual modulation, computed as the fold-change reduction in unpredictability from the 'No Context' to the 'Full Context' condition. The effect was feature-specific and strongest for low-level features (Conv2), providing a proof-of-concept that the inpainting-based modelling analysis, designed for natural stimuli, captures fundamental principles of contextual processing with simple stimuli too.*

We also performed a second “synthetic stimulus” analysis, varying not the amount of spatial context but the degree and type of mismatch. Using grating stimuli, we show that a mismatch in orientation primarily drives low-level unpredictability, whereas a textural disruption drives high-level unpredictability: between conv1 and conv5 we observe a gradual shift from being most sensitive to orientation disruption (shifted grating) towards most sensitive to textural disruption (phase noise).

Together with the example patches (e.g. natural textures showing high low-level unpredictability but low high-level unpredictability) this shows how the multi-level metric can meaningfully distinguish between violations of different image properties:

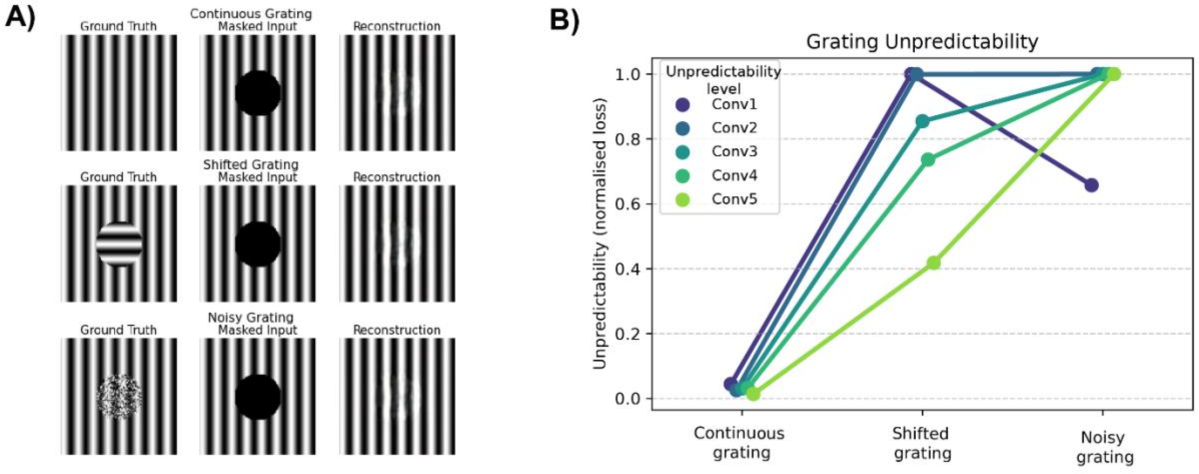

Figure R9 **Dissociating feature-level predictability with targeted synthetic mismatches.** This *analysis demonstrates how the multi-level unpredictability metric is sensitive to different feature violations (see Methods).* ***A)*** *We created three ground-truth stimuli where the surround was a continuous vertical grating. The central masked patch contained either a continuous grating (no mismatch), an orthogonal grating ('Shifted'), or a grating with phase noise ('Noisy'). Because the masked input to the inpainting model was identical in all cases, the model produced the same reconstruction: a continuous vertical grating.* ***B)*** *Unpredictability was quantified as the normalized content loss between each ground truth and the single reconstruction. This revealed a clear dissociation: low-level unpredictability (Conv1) was maximally driven by the orientation mismatch in the 'Shifted' condition. In contrast, high-level unpredictability (Conv5) was much less sensitive to the orientation shift (i.e. most orientation invariant) but strongly driven by the textural disruption in the 'Noisy' condition. This confirms that the metric can distinguish between violations of low-level higher-level image properties.*

Together, these new analyses confirm that our inpainting-based framework, while designed for the complexity of natural scenes, appears to be indeed sensitive to fundamental features of contextual processing established in classic visual neuroscience.

We are grateful for the reviewer's feedback, which has prompted these clarifications and additions.

> Relatedly, in classical studies with both parametric and natural stimuli, sensitivity to center-surround similarity is both stimulus-specific and neuron-specific (e.g. https://pubmed.ncbi.nlm.nih.gov/7477405/ ; https://pubmed.ncbi.nlm.nih.gov/12424293/). In contrast, this metric seems to be only image-specific, or feature layer specific in some analysis, except for the fact that it is computed by masking the image portion falling inside the RF of each neuron (if I got this right?). It is possible that a more granular estimation of predictability based on the per-neuron feature (not just the deep net layer) would reveal stronger sensitivity than the "perceptual" predictability for the entire image.

We thank the reviewer for this suggestion regarding neuron-specific -- and tuning-specific – predictability.

First, to clarify, our predictability metric is already computed on a per-neuron basis. For each of the 3,176 neurons in our dataset, we individually mask its unique receptive field across all 118 natural images and compute a distinct set of predictability estimates. This amounts to over 370,000 individual inpainting and predictability analyses, making the current approach already stimulus-specific and neuron-specific.

We agree that the granularity could be extended further by considering the interaction between predictability and the specific tuning properties of each neuron – for instance, by computing predictability specifically for a neuron's preferred orientation and spatial frequency. However, implementing such a 'tuning-contingent' predictability analysis presents significant technical and conceptual challenges that place it beyond the scope of the current study. Computationally, computing predictability not just for every receptive field but also for every potential neural tuning would require a major expansion of the analysis, and applying it for each of the >370,000 inpainted patches would be prohibitively costly. Moreover, defining such a metric is only straightforward for orientation. This would necessitate a departure from our broad-coverage approach – which analyzes all neurons with a well-defined receptive field – to a quite different, much more focused analysis on a subset of cells that are sharply tuned around orientation and/or spatial frequency. As such, we believe this extension would constitute a separate research project in its own right.

Nevertheless, we believe this is a fascinating direction for future research. We how highlight this as a limitation of our approach and avenue for future work in the discussion:

> *In our analysis, multiple predictability estimates are computed for each neuron's receptive field. However, this metric does not explicitly account for the specific tuning properties of each neuron. This is a limitation, because a rich body of work using artificial stimuli has established that contextual modulation in the visual cortex can be highly feature-specific, e.g. being strongest when the surround matches the neuron's preferred orientation (Knierim and Van Essen, 1992; Cavanaugh et al., 2002). Extending this principle to our framework would be a powerful next step. For an orientation-tuned neuron, one could assess not just the overall predictability of an image patch, but specifically the predictability of its preferred orientation content. Such a 'tuning-contingent' metric might reveal even stronger and more precise modulations. Furthermore, this feature-specific approach would provide a framework for modeling the nonlinear dynamics of contextual modulation, for instance, by allowing predictability to directly gate or weigh the divisive influence of surround normalization on a neuron's contrast response.*

We hope our publicly available code might facilitate future work in this direction.

> Another issue is that sensitivity to predictability is determined with a linear regression approach. Predict firing rate as linear combination of low-level features (e.g. contrast, orientation etc.) plus non-visual factors (e.g. running speed) and compare w/out predictability. This approach has become common (in other contexts), but it ignores that contextual effects in visual cortex are nonlinear and their nonlinearity has been formalized and characterized extensively (https://pubmed.ncbi.nlm.nih.gov/12424292/). So for instance the effects might differ if predictability were used to weigh the surround normalization term of E_norm in section 4.6.

We thank the reviewer for raising this point about the relationship between our regression modelling and the non-linearities of neural firing. We agree that the linear model is a simplification.

Our rationale for using a linear regression model is twofold. First, its primary purpose is to serve as a robust, large-scale statistical test. We are testing the core hypothesis that, on average, a monotonic relationship exists between unpredictability and neural firing. As long as this condition holds, a linear model is a powerful and direct tool for detecting the presence of such an effect, even if it does not capture its precise nonlinear form. The sheer scale of our analysis makes a linear approach the most feasible and choice for this broad-coverage investigation.

The reviewer's specific suggestion – that predictability could weigh the surround normalization term – is an excellent example of the tuning-contingent, nonlinear interactions that our current model does not capture. This relates to the points raised in the previous comment.

To make this reasoning clearer, we made two additions:

1. We have added a clarifying statement to the Methods (Section 4.7) regarding the scope and purpose of our linear modeling approach:

> *For each neuron and each timepoint, we fitted separate linear regression models, allowing us to track how the influence of predictability evolved over the visual response time course. We chose this linear modelling framework for its statistical power and interpretability in testing for a monotonic relationship at a large scale. This approach necessarily simplifies the known nonlinear dynamics of neuronal firing and some non-linear interactions observed in contextual modulation in V1. However, its purpose is to provide a robust and direct test for the presence and direction of the effect across the population, rather than to serve as a complete or mechanistic model.*
> *We fitted two separate types of models (…)*

2. We have included this specific suggestions in the new paragraph in the Discussion to explicitly link the idea of tuning-contingent predictability to the modeling of such nonlinear interactions:

> *Furthermore, this feature-specific approach would provide a natural framework for modeling the nonlinear dynamics of contextual modulation, for instance, by allowing predictability to directly gate or weigh the divisive influence of surround normalization on a neuron's contrast response.*

> 2. Effect size and timing
> The authors report modulation by unpredictability, i.e. higher firing rate for unpredictable images, suppressed firing rate for predictable images. This effect is smaller than modulation by low-level features, but significant. Nonetheless, the effect reported is quite small: Line 104 reports a DeltaR of 0.0036 over an R of approximately 0.2 (Fig. 2a,b), so a 5% effect. I understand it is statistically significant, but perhaps it is worth 1) understanding why it is stronger/weaker in some neurons than others (if indeed it is the case that the magnitude is variable across cases) and 2) speculate how such a small modulation could impact or be used by downstream computation.

We thank the reviewer for this important point regarding effect sizes.

First, we should clarify that, as we wrote in the main text, the ΔR metric should *not* be interpreted as a direct measure of effect size. It provides a highly conservative statistical test for the *presence* of an effect by isolating only the *unique* contribution, which in naturalistic paradigms is often a fraction of the total variance. However, the reviewer comment has motivated us to revisit the topic and we do agree it warrants a more detailed treatment. We have therefore added a new paragraph to the Discussion that explicitly addresses the complexities of interpreting effect sizes in our study, considering how follow-up work addressing feature correlations and the choice of generative model may lead to a better possibility to accurately the relative importance of spatial predictability:

> *Interpreting the magnitude of this effect, however, requires careful consideration. The use of natural scenes is both a strength and a limitation, as it introduces inherent correlations between spatial unpredictability and low-level features like local contrast. This results in a large portion of shared neural variance, meaning that metrics like ΔR, which isolate unique contributions, may systematically underestimate the relative importance of predictability. An exciting follow-up direction is therefore to use natural images specifically sampled to minimise these correlations (or to maximise the dissociation between different levels of predictability). Furthermore, our approach may underestimate predictability's importance because our metrics are derived from a general-purpose computer vision model, not one tailored to mouse vision. A model trained on a 'rodent visual diet' or a dataset using more ecologically valid stimuli could reveal a substantially larger effect, suggesting the estimates reported here may represent a conservative lower bound.*

We again would like to thank the reviewer for bringing this point to our attention.

> The key reference is Uran et al 2022 where macaque V1 was shown to be sensitive to predictability. Line 55 states that in that paper sensitivity emerges 200-600ms after stimulus onset, whereas here images are presented for 250ms only and the analysis appears to be restricted to that window. The authors could comment on the reasoning why these presumably feedback/high-level effects are expected within the shorter window than in Uran.

This is a misunderstanding, we apologize for not being sufficiently clear.

Uran et al. observed an overall predictability effect (i.e. with omnibus metric that of overall predictability) both in the early (50-150 ms) and in the late time window (200-600 ms). The comment about late firing rates only pertains to the multi-level unpredictability analysis, and the observed preference for higher-level predictability. In Uran et al., this analysis was simply not reported for early spiking so it is impossible to tell if our findings are different from theirs. We have now rewritten the phrase in the main text to emphasise that it is only this particular finding in which they focus on a different time-window

> *[They] found that less predictable patches evoked higher firing rates in macaque V1. Strikingly, in a later analysis focusing specifically on a later phase of processing (200-600 ms after stimulus onset) they found the unpredictability of high-level features of the image part drove activity most strongly.*

We again would like to thank the reviewer for spotting this.

> The timing of the effect is also in line with the expectation that predictability requires more recurrent processing than low-level features, but again the effect is small, a 6 ms difference. How reliable and meaningful is that difference, given that the analysis of rate is based on 10 ms counting windows with a 30-ms smoothing?

We appreciate the reviewer's point about the latency difference.

First, we should note that the latency difference is visually apparent in Figure 2d, where contrast energy effects emerge earlier than unpredictability effects. Our quantitative analysis was primarily based to rigorously characterizes this difference (intra-animal fractional peak latency with bootstraps across mice), yielding an average of 6 ms -- though mice show variable differences.

While this difference is directionally consistent with predictability involving recurrent processing, the modest average magnitude could indicate relatively local recurrence within or near V1, though the small effect size could also reflect the limited temporal sensitivity of our analysis (10 ms bins, and substantial inter-animal and inter-neuron variability of firing rate curves). Given these methodological limitations, temporal dynamics are not a primary focus of our study, and all key analyses employ temporal aggregation. We report these descriptive statistics on the latency primarily for completeness.

To address the reviewer's concern, we now more explicitly caution this result in the main text (line 240):

> *"Critically, the contrast effect appears to modulate the neural response earlier (Figure 2c-d; average latency to 50% peak: M = 71.0 ms, SE = [68.8, 73.2] vs. unpredictability: M = 77.3 ms, SE = [75.1, 79.6]), qualitatively consistent with predictability effects involving recurrent processing. However, this difference is subtle, and the small magnitude may indicate primarily local recurrent processing within or near primary visual cortex rather than long-range feedback. We note that the temporal resolution of our dataset (10 ms bins) and variability across animals make this analysis suboptimal for fine-grained latency distinctions."*

> One of the results I found most interesting is that the modulation by predictability is stronger for predictability defined by deep feature layers (presumably more closely related to perception) than low-level features. Conversely, the base encoding model relies more on low-level features. It would be interesting to know if these two effects also have different timecourse. For instance, does the timing of the effect of predictability vary when using superficial versus deep features to compute predictability?

We thank the reviewer for their appraisal and the suggestion.

We did indeed look into this but observed no clear differences. We did not report this earlier because the null-results were also not extremely convincing. For completeness we report it below.

First, focusing on V1 (the area with the best coverage and hence highest SNR), we visually compared the modulation functions (contrast versus multi-level unpredictability):

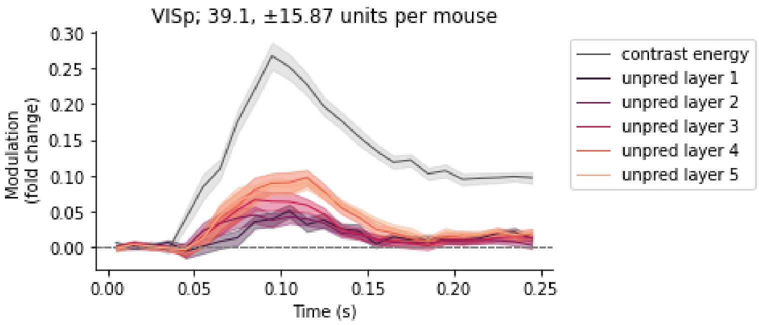

Figure R 10. As Figure 2D but for different unpredictability estimates

The latency difference between contrast and unpredictability appears fairly clear (although numerically, if we do rigorous paired latency estimation across mice, the difference is small). However, between different predictability effects, latency differences are less clear, and there are no statistically significant patterns observed:

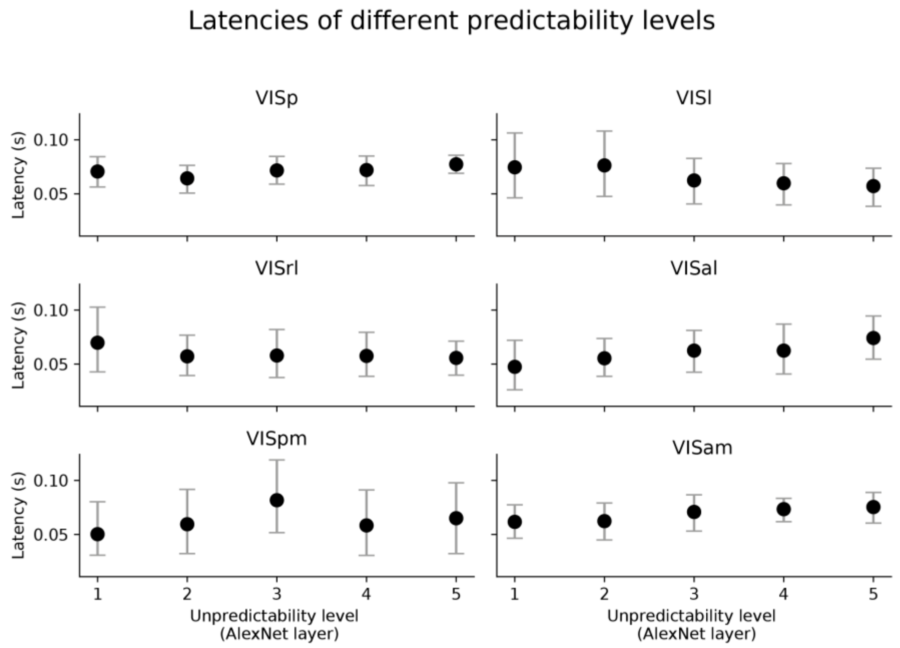

Figure R 11. Latencies across ROIs and predictability metrics. Big dots with error bars indicate mean latency (time to 50% of max), plus bootstrapped 95% confidence intervals across mice.

For transparency, we now report this Figure (Fig S…). However, we would prefer not to draw strong conclusions from this null-result because we think the dataset and analysis may simply not be ideally suited for detecting small latency differences.

> 3. The theory is underspecified
> The Discussion on the laminar specificity of the effects expected by predictive coding raises two issues. First, given that the qualitative effects based on the abstract theory are not clear cut, it seems important to implement the theory and conduct simulations to provide more granular predictions. And second, the discussion closes by suggesting that more granular data analysis would be required too, but does not give any hint of what those experiments and analysis would entail. Overall, the paper would be much stronger if it were driven by an actual implementation of the theory, to better understand what aspects of predictive coding are distinctively supported by the data.

We thank the reviewer for their thoughtful comments.

Regarding the need for theoretical simulations: We agree that this would be useful – which is why we say so in the discussion – but we respectfully note that this is beyond the scope of the current work.

Predictive processing is not a single, unified computational model or even theory but rather a family of theories with diverse implementations. Critically, we are not aware of any existing implementation of predictive processing (with separate error and prediction units) that can meaningfully process arbitrary natural images with specified receptive field geometries across thousands of neurons in our dataset. Developing such a model would require substantial theoretical work effectively constituting a full theoretical paper in itself.

Our contribution is fundamentally empirical – we operationalized sensory predictability (previously primarily studied with simple, artificial stimuli) and tested its influence at large scale across the mouse visual system using naturalistic stimuli. This required a substantial technical and computational undertaking that constitutes our primary contribution.

We acknowledge in our Discussion that the theoretical work the reviewer proposes would be very valuable for future studies, which is precisely why we explicitly state this as a limitation. We believe our empirical findings stand on their own as an advance in understanding spatial prediction, while appropriately acknowledging where theoretical extensions would be valuable for future work.

Regarding more granular analyses: We apologize for the ambiguity. The "more granular" analyses we envision are actually both more granular at the theoretical level (with simulations for the exact stimuli and receptive fields observed) and at the physiological level: (1) substantially larger numbers of simultaneously recorded neurons in both superficial and deep layers to increase statistical power, and (2) histological validation of laminar boundaries to more precisely classify neuron positions, as the current CCF-based classification introduces some uncertainty. We have now clarified this in the Discussion:

> *Therefore, while the laminar effect we observe is striking, strong conclusions about the existence of error/prediction units would require more granular analyses at both theoretical and physiological levels. Theoretically, simulations of predictive coding models using matched stimuli and receptive field configurations would provide more precise laminar predictions—though implementing such models for natural scenes remains an open challenge. Physiologically, larger samples of simultaneously recorded neurons per layer and histological validation of laminar boundaries would reduce the CCF-based classification uncertainty and increase statistical power.*

> 4. Additional comments and suggestions
> The author could comment on the findings by Tolias group that suggest V1 selectivity is more complex, i.e. tuned to higher level features, than classically thought. This seems somewhat relevant to the dissociation reported here between sensitivity to low-level features versus predictability. https://pubmed.ncbi.nlm.nih.gov/31686023/

Thank you, that is a great addition, we now cite this work in the discussion.

> Surround modulation has been extensively linked to predictive coding. It seems appropriate to discuss the relation to that work. E.g. https://pubmed.ncbi.nlm.nih.gov/21315102/

Thank you, we now cite this alongside other works explaining surrounding suppression via redundancy reduction and/or prediction error computation.

Methods 4.4: the loss function includes multiple terms, with specific choices of their relative weights. It would be helpful to write down the key equations and explain them, so the reader doesn't need to hunt them down in the referred papers.

We have added the loss function equation with specific hyperparameter values. As we use Liu et al.'s implementation without any modifications beyond specific hyperparameters, we reference their paper for complete mathematical derivations to maintain proportionality in our methods section.

Methods 4.5: what is the rationale for using Alex Net for the predictability analysis while the model uses VGG? How do we know that does not impact the results?

We thank the reviewer for noticing and asking. This was a deliberate choice reflecting two distinct constraints in our methodology. For the neural data analysis based on predictability, we had to use a single model for both predictability and encoding to ensure a direct comparison (Fig 3), and based on prior work (in particular by Nayebi) on encoding modelling for mouse visual cortex, we chose AlexNet because it performs best to predict mouse visual cortical responses, presumably because it is more shallow. In contrast, model fine-tuning, we re-used the VGG-based perceptual loss to maintain consistency with the pre-training of the in-painting architecture. However, for the effect it does not matter – in fact, Uran et al. used VGG for both.

Fig 2 caption says '0-30 ms' but methods says 20 ms?

Thank you for catching this, methods (20ms) were correct. Fixed

Line 331, sentence starting with "Moreover" is repeated twice.
Line 336, define CCF.
Line 357 'receptor-field' is it 'receptive-field' instead?
Line 380 'L2' not properly formatted
Line 426 'this set comprehensive set' remove the first 'set'
Line 472 missing full stop before 'Each'

Thanks, all typo's fixed.
